# Beyond antibiotic prescribing rates: first-line antibiotic selection, prescription duration, and associated factors for respiratory encounters in urgent care

**DOI:** 10.1017/ash.2023.416

**Published:** 2023-09-05

**Authors:** Allan M. Seibert, Carly Schenk, Whitney R. Buckel, Payal K. Patel, Nora Fino, Valoree Stanfield, Adam L. Hersh, Eddie Stenehjem

**Affiliations:** 1 Division of Infectious Diseases, Intermountain Health, Salt Lake City, UT, USA; 2 Maine Medical Center, Portland, ME, USA; 3 Pharmacy Services, Intermountain Health, Salt Lake City, UT, USA; 4 Division of Epidemiology, Department of Internal Medicine, University of Utah, Salt Lake City, UT, USA; 5 Office of Patient Experience, Intermountain Health, Salt Lake City, UT, USA; 6 Department of Pediatrics, Division of Infectious Diseases, University of Utah School of Medicine, Salt Lake City, UT, USA

**Keywords:** urgent care, antibiotic stewardship, antibiotic selection, prescription duration, respiratory tract infections

## Abstract

**Objective:**

Assess urgent care (UC) clinician prescribing practices and factors associated with first-line antibiotic selection and recommended duration of therapy for sinusitis, acute otitis media (AOM), and pharyngitis.

**Design:**

Retrospective cohort study.

**Participants:**

All respiratory UC encounters and clinicians in the Intermountain Health (IH) network, July 1st, 2019–June 30th, 2020.

**Methods:**

Descriptive statistics were used to characterize first-line antibiotic selection rates and the duration of antibiotic prescriptions during pharyngitis, sinusitis, and AOM UC encounters. Patient and clinician characteristics were evaluated. System-specific guidelines recommended 5–10 days of penicillin, amoxicillin, or amoxicillin-clavulanate as first-line. Alternative therapies were recommended for penicillin allergy. Generalized estimating equation modeling was used to assess predictors of first-line antibiotic selection, prescription duration, and first-line antibiotic prescriptions for an appropriate duration.

**Results:**

Among encounters in which an antibiotic was prescribed, the rate of first-line antibiotic selection was 75%, the recommended duration was 70%, and the rate of first-line antibiotic selection for the recommended duration was 53%. AOM was associated with the highest rate of first-line prescriptions (83%); sinusitis the lowest (69%). Pharyngitis was associated with the highest rate of prescriptions for the recommended duration (91%); AOM the lowest (51%). Penicillin allergy was the strongest predictor of non–first-line selection (OR = 0.02, 95% CI [0.02, 0.02]) and was also associated with extended duration prescriptions (OR = 0.87 [0.80, 0.95]).

**Conclusions:**

First-line antibiotic selection and duration for respiratory UC encounters varied by diagnosis and patient characteristics. These areas can serve as a focus for ongoing stewardship efforts.

## Introduction

Most antibiotic use originates in the ambulatory setting and an estimated one-third of outpatient antibiotic prescriptions are thought to be unnecessary.^1,2^ The rate of first-line antibiotic use in outpatient settings, including emergency departments and retail/urgent care clinics, has been reported as approximately 50%: lower than national goals even after accounting for treatment failures and documented penicillin allergy.^3,4^ Antimicrobial stewardship efforts often prioritize the decision of whether or not to prescribe an antibiotic for a given condition along with optimizing antibiotic selection. Duration of therapy is also an important antibiotic stewardship target and inappropriate antibiotic prescription durations have been described for a variety of outpatient conditions.^5,6^ Nationally, in all age groups, pharyngitis, sinusitis, and acute otitis media (AOM) account for over 50% of all outpatient respiratory antibiotic prescriptions and this figure is over 70% in children,^2^ highlighting the need to focus ambulatory stewardship efforts on respiratory encounters. Additionally, there have been calls to reduce antibiotic use by decreasing the duration of therapy for sinusitis and AOM.^5,7–11^

Urgent care (UC) centers have been one of the fastest-growing settings for outpatient care delivery in the United States (US).^12,13^ Since 2019, UC centers have exhibited a 60% increase in patient volume, and this trend is forecast to continue in the coming years.^14^ Compared to other outpatient settings, UC encounters result in more overall and unnecessary antibiotic prescriptions.^15^ Respiratory tract infections, which are often associated with inappropriate antibiotic prescribing, are among the most common types of diagnoses evaluated in UC.^16^ While some studies have previously evaluated antibiotic selection and duration separately and in tandem for infectious conditions managed in pediatric UC settings, further understanding of these choices for UC sites that serve both adult and pediatric populations is needed.^5,7,17–20^

We have previously shown the impact of a multifaceted antibiotic stewardship intervention on reducing outpatient antibiotic prescriptions for respiratory UC encounters in a large integrated healthcare system.^21^ During this intervention, the Intermountain Health (IH) UC network reduced antibiotic prescribing in respiratory encounters from 48% to 33% over a 1-year period. Herein, we describe further opportunities for improvement by examining respiratory UC encounters where an antibiotic was prescribed during this period of decreased antibiotic prescribing. We assessed clinician prescribing practices and adherence to first-line antibiotic selection and duration of therapy recommended by institution-specific evidence-based guidelines for pharyngitis, sinusitis, and AOM. We also sought to identify factors associated with first-line and non–first-line antibiotic selection and extended-duration prescriptions to guide future stewardship efforts across our UC network.

## Methods

This was a retrospective cohort study of pharyngitis, sinusitis, and AOM encounters in the IH UC network from July 1st, 2019 through June 30th, 2020. IH is a nonprofit, integrated, healthcare delivery system in the Mountain West and during the study period operated 38 UC clinics, which included 32 providing care for patients of all ages (“InstaCare”) and six providing care exclusively to children fewer than 18 years old (“KidsCare”). InstaCare is staffed predominantly by family medicine physicians while KidsCare is exclusively staffed by pediatricians. Pharyngitis (both streptococcal pharyngitis and unspecified pharyngitis), sinusitis, and AOM UC encounters were identified using a validated methodology based on the *International Classification of Diseases, 10*th *Revision, Clinical Modification* (ICD10) codes.^16^ We excluded clinicians and their encounters if they had fewer than 25 UC encounters during the study.^21^ Additionally, encounters with missing provider information were excluded. Sinusitis, pharyngitis, and AOM encounters in which an antibiotic was prescribed were assessed for first-line antibiotic selection and appropriate duration of therapy based on system-specific evidence-based guidelines using orders captured via the electronic health record (EHR). Prescription information, including the drug name and the duration of the prescription, was electronically extracted from the record. Antibiotic allergies were identified in the EHR along with patient demographic data. Race, ethnicity, and gender data are indicated by patients during the registration process for all encounters that occur throughout the IH system. Clinicians were characterized as either an MD/DO clinician or an advanced practice clinician (APC) (ie, a nurse practitioner or physician assistant (NP/PA)).

First-line therapy for pharyngitis was penicillin or amoxicillin and the recommended duration was 10 days. In patients with acute sinusitis, amoxicillin or amoxicillin-clavulanate depending on disease severity was considered first line and the recommended duration was 7 days in adults and 10 days in pediatric patients. In patients with AOM, amoxicillin or amoxicillin-clavulanate was the first-line therapy. For patients with AOM younger than 2 years old and those with severe AOM, the recommended duration was 10 days and 5–7 days in all other patients. In patients with a penicillin allergy, alternative second-line antibiotics were recommended. The recommended duration of therapy for penicillin-allergic patients receiving second-line antibiotics was the same as patients receiving first-line therapy except for those receiving azithromycin for pharyngitis or ceftriaxone for AOM. Because we were unable to assess disease severity in AOM, the shortest age-specific recommended duration (eg, 5–7 days in patients >2 years old) was considered appropriate in AOM encounters. Prescriptions exceeding the recommended duration were considered inappropriate while those equal to or less than the recommended duration were considered appropriate. Institution-specific guideline-recommended first and alternative second-line antibiotics for the conditions studied along with recommended durations for prescriptions are summarized in Table 1.

**Table 1.** Institution-specific guidelines recommended first-line and second-line antibiotics and duration of therapy for pharyngitis, acute sinusitis, and acute otitis media

Respiratory condition	First-line therapy	Recommended duration (1st line)	Alternative second-line therapy	Recommended duration (2nd line)
Pharyngitis	Penicillin	10 d	Cephalexin	10 d
	Amoxicillin		Clindamycin	10 d
			Azithromycin	3 d
Acute sinusitis	Amoxicillin	7 d^a^	Cefdinir OR Doxycycline (>18 yr old)	7 d
	Amoxicillin/clavulanate		Cefdinir AND Clindamycin (≤18 yr old)	
Acute otitis media^b^	Amoxicillin	<2 yr – 10 d	Cefdinir	<2 yr – 10 d
	Amoxicillin/clavulanate	≥2 yr – 5–7 d	Clindamycin	≥2 yr – 5–7 d
			Ceftriaxone	Ceftriaxone: 3 d

a 10 d of therapy was recommended for pediatric acute sinusitis.

b For severe acute otitis media, 10 d of therapy was recommended.

Descriptive statistics were used to characterize first-line antibiotic selection, appropriateness of duration of antibiotic prescriptions, and a combined outcome of first-line antibiotic prescriptions for an appropriate duration across each condition as well as provider (MD/DO vs APC) and clinic type (InstaCare vs KidsCare). To examine factors associated with first-line antibiotic prescriptions, appropriate duration prescriptions, and a combined outcome of first-line antibiotic prescriptions for an appropriate duration, we applied univariate and multivariable generalized estimating equation (GEE) models. GEE models allow for the analysis of correlated data by estimating the population-averaged effects of covariates on the outcome variables. Models here accounted for correlation within providers and multivariable models were adjusted for all factors examined.^22^ A *P*-value of <.05 was considered statistically significant. The Institutional Review Board of Intermountain Health approved this study and waived informed consent.

## Results

During the study period, 98,510 encounters for sinusitis, AOM, or pharyngitis were identified. Of these, 94,228 met inclusion criteria and represented 83,886 unique patients which were seen by 270 clinicians. There were 27,601 (29.3%) encounters for sinusitis, 13,743 (14.6%) for AOM, and 52,884 (56.1%) for pharyngitis. A total of 54,072 (57.4%) encounters resulted in an antibiotic prescription, with AOM (94.5%) demonstrating the highest antibiotic prescription rate followed by sinusitis (84.3%), and pharyngitis (33.7%). For the antibiotic-treated population, the mean age of patients was 29.4 (SD 19.9) with 33.1% of patients ≤ 18 years old **(**Table 2 **)**. 31,558 (58.4%) encounters occurred for female patients, 50,437 (93.7%) encounters were for White patients, and 47,690 (90.1%) occurred in non-Hispanic patients. 76.4% of clinicians were MD/DO and 23.6% were APC (NP/PA) clinicians. The majority occurred in InstaCare clinics (93.5%). Penicillin allergy was documented in 6,380 (11.8%) encounters in which an antibiotic was prescribed. The most commonly prescribed antibiotics for the included conditions were amoxicillin (61.5%), doxycycline (9.9%), amoxicillin-clavulanate (8.9%), cefdinir (7.3%), and penicillin VK (4.0%). Most patients received an oral antibiotic prescription, with only 1,092 (2.0%) patients receiving a parenteral antibiotic, and most of these (*n* = 887) were for pharyngitis. Patient and provider characteristics for all encounters associated with an antibiotic prescription are presented in Table 2.

**Table 2.** Patient and provider characteristics for all encounters with an antibiotic prescription (Abx)

Characteristic	Overall *N* = 54,072	Antibiotic selection		Antibiotic duration		First-line prescription for appropriate duration (*n* = 28,655)
		Did not receive first-line Abx (*n* = 13,785)	Received first-line Abx (*n* = 40,287)	Inappropriate duration (*n* = 16,373)	Appropriate duration (*n* = 37,699)	
Diagnosis, *N* (%)						
Pharyngitis	17,824 (33.0%)	4,341 (24.4%)	13,483 (75.6%)	1,683 (9.4%)	16,141 (90.6%)	12,455 (69.9%)
Sinusitis	23,254 (43.0%)	7,238 (31.1%)	16,016 (68.9%)	8,361 (36.0%)	14,893 (64.0%)	10,526 (45.3%)
Otitis media	12,994 (24.0%)	2,206 (17.0%)	10,788 (83.0%)	6,329 (48.7%)	6,665 (51.3%)	5,674 (43.7%)
Age, years, mean (SD)	29.4 (19.9)	34.8 (20.1)	27.6 (19.6)	30.2 (21.4)	29.1 (19.3)	27.5 (19.0)
Age group, *N* (%)						
0–2	2,313 (4.3%)	348 (15.0%)	1,965 (85.0%)	131 (5.7%)	2,182 (94.3%)	1,882 (81.4%)
2–18	15,573 (28.8%)	2,721 (17.5%)	12,852 (82.5%)	5,623 (36.1%)	9,950 (63.9%)	8,258 (53.0%)
18.1–64	33,134 (61.3%)	9,552 (28.8%)	23,582 (71.2%)	9,403 (28.4%)	23,731 (71.6%)	17,323 (52.3%)
65+	3,052 (5.6%)	1,164 (38.1%)	1,888 (61.9%)	1,216 (39.8%)	1,836 (60.2%)	1,192 (39.1%)
Female, *N* (%)						
No	22,510 (41.6%)	5,363 (23.8%)	17,147 (76.2%)	7,060 (31.4%)	15,450 (68.6%)	11,988 (53.3%)
Yes	31,558 (58.4%)	8,422 (26.7%)	23,136 (73.3%)	9,312 (29.5%)	22,246 (70.5%)	16,664 (52.8%)
White race, *N* (%)						
No	3,399 (6.3%)	684 (20.1%)	2,715 (79.9%)	930 (27.4%)	2,469 (72.6%)	1,981 (58.3%)
Yes	50,437 (93.7%)	13,042 (25.9%)	37,395 (74.1%)	15,415 (30.6%)	35,022 (69.4%)	26,517 (52.6%)
Hispanic ethnicity, *N* (%)						
No	47,690 (90.1%)	12,338 (25.9%)	35,352 (74.1%)	14,592 (30.6%)	33,098 (69.4%)	25,061 (52.5%)
Yes	5,265 (9.9%)	1,188 (22.6%)	4,077 (77.4%)	1,514 (28.8%)	3,751 (71.2%)	2,925 (55.6%)
Clinic type, *N* (%)						
InstaCare	50,540 (93.5%)	13,312 (26.3%)	37,228 (73.7%)	15,661 (31.0%)	34,879 (69.0%)	26,172 (51.8%)
KidsCare	3,532 (6.5%)	473 (13.4%)	3,059 (86.6%)	712 (20.2%)	2,820 (79.8%)	2,483 (70.3%)
Provider type, *N* (%)						
MD/DO	41,292 (76.4%)	10,348 (25.1%)	30,944 (74.9%)	12,731 (30.8%)	28,561 (69.2%)	21,785 (52.8%)
APC (NP/PA)^a^	12,780 (23.6%)	3,437 (26.9%)	9,343 (73.1%)	3,642 (28.5%)	9,138 (71.5%)	6,870 (53.8%)
Penicillin allergy, *N* (%)						
No	47,692 (88.2%)	7,950 (16.7%)	39,742 (83.3%)	14,245 (29.9%)	33,447 (70.1%)	28,263 (59.3%)
Yes	6,380 (11.8%)	5,835 (91.5%)	545 (8.5%)	2,128 (33.4%)	4,252 (66.6%)	392 (6.1%)

a APC (NP/PA): Advanced practice clinician (nurse practitioner/physician assistant).

Overall, among encounters where an antibiotic was received, the rate of first-line antibiotic prescriptions was 74.5%. Sinusitis encounters received first-line therapy in 16,016 (68.9%) encounters, pharyngitis in 13,483 (75.6%) encounters, and AOM received first-line antibiotic therapy in 10,788 (83%) encounters. An appropriate duration was prescribed in 37,699 (69.7%) encounters. 6,665 (51.3%) AOM encounter prescriptions were for an appropriate duration compared to 14,893 (64%) sinusitis and 16,141 (90.6%) pharyngitis. Only 28,655 (53.0%) encounters received a first-line antibiotic for an appropriate duration. This varied by condition: 5,674 (43.7%) AOM encounters, 10,526 (45.3%) sinusitis encounters, and 12,455 (69.9%) pharyngitis encounters. MD/DO and APCs had similar rates of first-line antibiotic prescriptions for an appropriate duration (52.8% vs 53.8%). Among encounters with a documented penicillin allergy, 545 (8.5%) received a first-line antibiotic prescription compared to 39,742 (83.3%) without a documented penicillin allergy. Among those with a penicillin allergy, 4,771 (74.8%) received a recommended second-line agent and 1,064 (16.7%) received neither a first- nor second-line agent. The 5,835 encounters with a documented penicillin allergy that did not receive first-line therapy accounted for 42.3% (5,835/13,785) of all encounters that did not receive first-line therapy.

## Modeling

Univariate and multivariable GEE modeling, while accounting for correlation within providers, was performed to examine factors associated with first-line prescriptions and appropriate duration **(**Table 3 **)**. Sinusitis (OR = 0.56, 95% CI [0.47, 0.68]) and pharyngitis (OR = 0.58, 95% CI [0.48, 0.69]) encounters were less likely to receive a first-line antibiotic prescription compared to AOM but AOM encounters were also least likely to receive a prescription for an appropriate duration (OR = 0.11, 95% CI [0.08, 0.14]) compared to pharyngitis. Older patients were less likely to receive a first-line antibiotic prescription, per 1-year increase in age (OR = 0.99, 95% CI [0.98, 0.99]) but no association was identified between appropriate duration prescriptions and age (OR = 1.00, 95% CI [1.00, 1.00]). Female patients were more likely to receive a first-line antibiotic prescription (OR = 1.07, 95% CI [1.01, 1.13]) as well as prescriptions for an appropriate duration (OR = 1.03, 95% CI [1.00, 1.07]). Compared to non-White patients, White patients were less likely to receive a first-line antibiotic prescription (OR 0.85, 95% CI [0.75, 0.95]) or an antibiotic prescription for an appropriate duration (OR = 0.90, 95% CI [0.84, 0.97]). Non-Hispanic and Hispanic patients were similarly likely to receive a first-line antibiotic prescription however non-Hispanic patients were less likely to receive an antibiotic prescription for an appropriate duration (OR = 0.93, 95% CI [0.87, 0.99]). KidsCare UC centers were more likely to provide an antibiotic prescription for an appropriate duration (OR = 1.66, 95% CI [1.03, 2.66] and no significant differences were noted between multivariable modeling results for provider type categories across clinic types. Compared to patients without a documented penicillin allergy, those with allergy were less likely to receive a first-line antibiotic prescription (OR = 0.02, 95% CI [0.02, 0.02]) or a prescription for an appropriate duration (OR = 0.87, 95% CI [0.80, 0.95]).

**Table 3.** Results from GEE models accounting for correlation within provider for first-line prescribing and appropriate duration for all encounters with an antibiotic. Univariate and multivariable models are presented; multivariable models are adjusted for all factors listed in the table.

Characteristic	Odds ratios for first-line prescribing				Odds ratios for Appropriate Duration			
	Univariate models		Multivariable model		Univariate models		Multivariable model	
	Odds ratios (95% CI)	*P* value	Odds ratios (95% CI)	*P* value	Odds ratios (95% CI)	*P* value	Odds ratios (95% CI)	*P* value
Diagnosis, *N* (%)								
Pharyngitis	0.65 (0.57, 0.74)	<.001	0.58 (0.48, 0.69)	<.001	Ref		Ref	
Sinusitis	0.47 (0.41, 0.54)	<.001	0.56 (0.47, 0.68)	<.001	0.17 (0.12, 0.22)	<.001	0.17 (0.13, 0.23)	<.001
Otitis media	Ref		Ref		0.11 (0.09, 0.15)	<.001	0.11 (0.08, 0.14)	<.001
Age, per 1-yr increase	0.98 (0.98, 0.99)	<.001	0.99 (0.98, 0.99)	<.001	1.00 (0.99, 1.00)	.019	1.00 (1.00, 1.00)	.222
Female, *N* (%)								
No	Ref		Ref		Ref		Ref	
Yes	0.87 (0.83, 0.92)	<.001	1.07 (1.01, 1.13)	.031	1.06 (1.03, 1.10)	<.001	1.03 (1.00, 1.07)	.034
White race, *N* (%)								
No	Ref		Ref		Ref		Ref	
Yes	0.71 (0.65, 0.78)	<.001	0.85 (0.75, 0.95)	.005	0.88 (0.83, 0.95)	<.001	0.90 (0.84, 0.97)	.009
Hispanic ethnicity, *N* (%)								
No	0.83 (0.78, 0.89)	<.001	1.02 (0.94, 1.11)	.596	0.88 (0.82, 0.94)	<.001	0.93 (0.87, 0.99)	.021
Yes	Ref		Ref		Ref		Ref	
Clinic type, *N* (%)								
InstaCare	Ref		Ref		Ref		Ref	
KidsCare	1.62 (1.36, 1.91)	<.001	1.27 (0.99, 1.63)	.059	1.13 (0.90, 1.42)	.287	1.66 (1.03, 2.66)	.037
Provider type, *N* (%)								
MD/DO	Ref		Ref		Ref		Ref	
APC (NP/PA)^a^	0.89 (0.76, 1.05)	.181	1.24 (0.87, 1.78)	.233	0.96 (0.74, 1.26)	.772	0.87 (0.41, 1.87)	.727
Penicillin allergy, *N* (%)								
No	Ref		Ref		Ref		Ref	
Yes	0.02 (0.02, 0.02)	<.001	0.02 (0.02, 0.02)	<.001	0.84 (0.78, 0.90)	<.001	0.87 (0.80, 0.95)	.001

a APC (NP/PA): Advanced practice clinician (nurse practitioner/physician assistant).

GEE modeling was also applied to examine factors associated with first-line antibiotic prescriptions for an appropriate duration **(**Table 4 **)**. Among diagnosis groups, pharyngitis encounters were most likely to have prescriptions fulfilling both criteria (OR = 3.76, 95% CI [3.04, 4.64]) while AOM encounters were least likely. With each 1-year increase in age, first-line antibiotic prescriptions for an appropriate duration became less likely (OR = 0.99, 95% CI[0.99, 1.00]). Female patients (OR = 1.05, 95% CI [1.02, 1.09]) and encounters at KidsCare UC centers (OR = 1.33, 95% CI [1.01, 1.75]) were more likely to receive first-line antibiotic prescriptions for an appropriate duration. Compared to encounters without a penicillin allergy, those with a penicillin allergy were less likely to receive a first-line antibiotic prescription for an appropriate duration (OR = 0.03, 95% CI [0.03, 0.04]).

**Table 4.** Results from GEE models accounting for correlation within provider for first-line prescribing for appropriate duration for all encounters with an antibiotic. Univariate and multivariable models are presented; multivariable models are adjusted for all factors listed in the table.

Characteristic	First-line prescription for appropriate duration			
	Univariate models		Multivariable model	
	Odds ratios (95% CI)	*P* value	Odds ratios (95% CI)	*P* value
Diagnosis, *N* (%)				
Pharyngitis	3.04 (2.53, 3.66)	3.04 (2.53, 3.66)	3.76 (3.04, 4.64)	<.001
Sinusitis	1.04 (0.89, 1.23)	1.04 (0.89, 1.23)	1.34 (1.11, 1.62)	<.001
Otitis media	Ref		Ref	
Age, per 1-year increase	0.99 (0.99, 0.99)	<.001	0.99 (0.99, 1.00)	<.001
Female, *N* (%)				
No	Ref		Ref	
Yes	0.98 (0.94, 1.01)	.142	1.05 (1.02, 1.09)	.003
White race, *N* (%)				
No	Ref		Ref	
Yes	0.81 (0.76, 0.86)	<.001	0.91 (0.85, 0.97)	.006
Hispanic ethnicity, *N* (%)				
No	Ref		Ref	
Yes	0.87 (0.82, 0.92)	<.0001	0.99 (0.93, 1.05)	.659
Clinic type, *N* (%)				
InstaCare	Ref		Ref	
KidsCare	1.38 (1.16, 1.64)	<.001	1.33 (1.01, 1.75)	.044
Provider type, *N* (%)				
MD/DO	Ref		Ref	
APC (NP/PA)^a^	0.92 (0.76, 1.13)	.4322	1.13 (0.74, 1.74)	.564
Penicillin allergy, *N* (%)				
No	Ref		Ref	
Yes	0.05 (0.04, 0.06)	<.001	0.03 (0.03, 0.04)	<.001

a APC (NP/PA): Advanced practice clinician (nurse practitioner/physician assistant).

## Discussion

While 75% of encounters in which an antibiotic was prescribed were associated with a first-line antibiotic and 70% with an appropriate duration, only 53% of encounters were associated with a first-line antibiotic prescription for an appropriate duration. This indicates substantial opportunity to improve respiratory UC antibiotic prescribing beyond the decision to prescribe an antibiotic. Our analysis also revealed differences in first-line prescribing across age, gender, and race and differences in appropriate duration between gender, race, and ethnicity.

Unsurprisingly, a documented penicillin allergy impacted first-line antibiotic selection. In our study, penicillin allergy conferred the highest risk of receiving a non–first-line antibiotic. Collectively, patients with a documented penicillin allergy only accounted for 11.8% of encounters in which an antibiotic was prescribed, but more than 40% of non–first-line prescriptions. This suggests that delabeling penicillin allergies, many of which are inaccurate for a variety of reasons,^23–25^ could have a large effect on improving outpatient prescribing as previously reported.^26^ Interestingly, in our study 545 (8.5%) encounters with penicillin allergy received first-line therapy suggesting that some clinicians may have questioned the veracity of the documented allergy. Penicillin allergy also conferred a higher likelihood of an extended duration prescription despite guidelines suggesting similar durations of therapy for most alternative second-line agents recommended for penicillin-allergic patients. Further study is needed to understand this unexpected finding. One potential explanation could be that certain clinical characteristics differ between patients with and without allergy which in turn lead clinicians to treat penicillin-allergic patients with longer antibiotic courses. The differences we observed do not appear to be entirely related to allergy prevalence: penicillin allergy in non-White patients receiving an antibiotic was 8.3% compared to 12.0% of White patients. Prior studies have demonstrated higher rates of penicillin and beta-lactam allergy in adult and pediatric White patients.^27,28^ The rate of penicillin allergy in our overall population was 11.6%, similar to that of the general population.^29,30^

Older patients were less likely to receive a first-line agent or a first-line agent for an appropriate duration. While patients ≥65 years old represented approximately 5% of our study population, only 39.1% of encounters in this age category received a first-line antibiotic for an appropriate duration.^31^ Despite elderly patients being a small proportion of our population, these findings highlight the opportunity to further improve antibiotic prescribing in these patients. Our findings for pediatric patients, supported by age and clinic-type data, are consistent with existing literature.^31^ Gender and race were predictors for first-line antibiotic selection along with appropriate duration prescriptions, and ethnicity was also associated with appropriate prescription duration. There may be differences in patient expectations or demands not captured by our study contributing to these findings.

Some studies suggest higher prescribing rates by APCs^32^ while others demonstrate similar prescribing rates for MD/DO and APC providers.^33,34^ We identified similar practices for both clinician types. While it was not a primary or secondary objective of our study, we noted similar interprovider variability for measured outcomes among both provider types (Supplementary Material, Figure 1). This finding could reflect the IH UC system involving all clinicians in education, tracking, and an organizational commitment to stewardship efforts. Both MD/DO and APC providers participate equally in education and tracking efforts and receive the same academic detailing communications. Recent data suggest shorter courses of therapy are sufficient for sinusitis and AOM in certain situations,^10,35^ and our findings emphasize the opportunity to improve the duration of antibiotic prescriptions among all clinicians.

Overall, the rate of first-line prescribing remained less than 80%. 80% has been proposed as the target rate for first-line antibiotic prescribing, providing some margin for when non–first-line therapy may be appropriate due to first-line failure and penicillin allergy.^36–38^ Despite institutional guidelines suggesting durations of 5–7 days for sinusitis and AOM, encounters for penicillin-allergic patients were associated with a longer-duration prescription. These findings provide an opportunity to emphasize and reinforce first-line selection while also providing education regarding the appropriate duration of antibiotic therapy.

While some of our study encompassed the COVID-19 pandemic, we did not observe an impact on antibiotic selection or prescription duration. Despite an increase in azithromycin use nationwide, we noted a decrease in azithromycin use from 4.5% in July 2019 to 1.2% in June 2020 and during March 2020–June 2020, azithromycin prescribing rates for respiratory UC encounters in our system remained less than 3%.^21^ Our study has limitations. First, we relied solely on ICD10 codes and did not assess for concordance between coding and the clinical diagnosis despite using a validated methodology. Second, we defined the appropriate duration for AOM in patients older than 2 years of age as 7 days or less while our institutional guideline recommends 10 days of therapy for severe AOM. However, our data extraction methods were unable to account for severity which could have led to an underestimation of appropriate duration prescriptions. Similarly, for our analysis, we defined the appropriate duration for sinusitis as 7 days when our pediatric guideline recommended 10 days, though efforts are underway to update this guideline to recommend a shorter duration. The rates of sinusitis in the pediatric population were low (7.7%), but this also may have underestimated the rate of appropriate duration prescriptions. Patient-preferred language, geography, and socioeconomic variables not assessed could have created a differential impact on access to care or expression of expectations and thus affected antibiotic selection and prescription duration as well. We did not examine what percentage of non–first-line therapy was due to failure or adverse events on first-line antibiotics. However, 1,353 (2.5%) of all encounters with an antibiotic occurred within 30 days of a previous encounter with an antibiotic prescription. Of these, 528 (39.1%) received a first-line antibiotic. For these second antibiotic-associated encounters, we were unable to capture if the prescription associated with the initial encounter was filled, or how much of the initial prescription was consumed. While we did not exclude encounters occurring within 30 days of an initial encounter, the first-line prescribing rate for encounters where there was not a subsequent antibiotic-associated encounter within 30 days was 75.4%, similar to our findings when examining all antibiotic-associated encounters. Lastly, we did not examine the appropriateness of antibiotic initiation and it is possible a proportion of prescriptions were not indicated.

Even in large vertically integrated healthcare systems with robust stewardship programs and amidst sustainable interventions that reduce respiratory antibiotic prescribing,^21^ there exist significant opportunities to improve prescribing practices beyond the *Antibiotic Use for Respiratory Conditions* Healthcare Effectiveness Data and Information Set (HEDIS) metric.^39^ Our findings emphasize the importance of delabeling antibiotic allergies when feasible and appropriate. Healthcare systems should also consider strategies such as prospective audit and feedback and peer comparison dashboards to improve the duration of antibiotic prescriptions given the success of these approaches in other areas of outpatient stewardship.^40^ First-line antibiotic prescriptions for an appropriate duration could also be a useful measure to evaluate antibiotic use for conditions where antibiotics may or may not be indicated while also being actionable and relevant to clinicians. Optimizing prescriptions for first-line antibiotics should not be considered separate from optimizing the duration of therapy: both are key components of antibiotic stewardship and a dual focus on antibiotic selection and duration should be considered as antibiotic stewardship interventions and metrics are further developed.

## Supporting information

Seibert et al. supplementary material Seibert et al. supplementary material

## Supplementary material

For supplementary material accompanying this paper visit http://doi.org/10.1017/ash.2023.416. click here to view supplementary material
