# Supplementary material for: Beyond antibiotic prescribing rates: first-line antibiotic selection, prescription duration, and associated factors for respiratory encounters in urgent care
Source: Antimicrob Steward Healthc Epidemiol. 2023 Sep 5;3(1):e146. doi: 10.1017/ash.2023.416 (PMC10523551; doi:10.1017/ash.2023.416)
Supplement: Seibert et al. supplementary material [file S2732494X23004163sup001.docx]

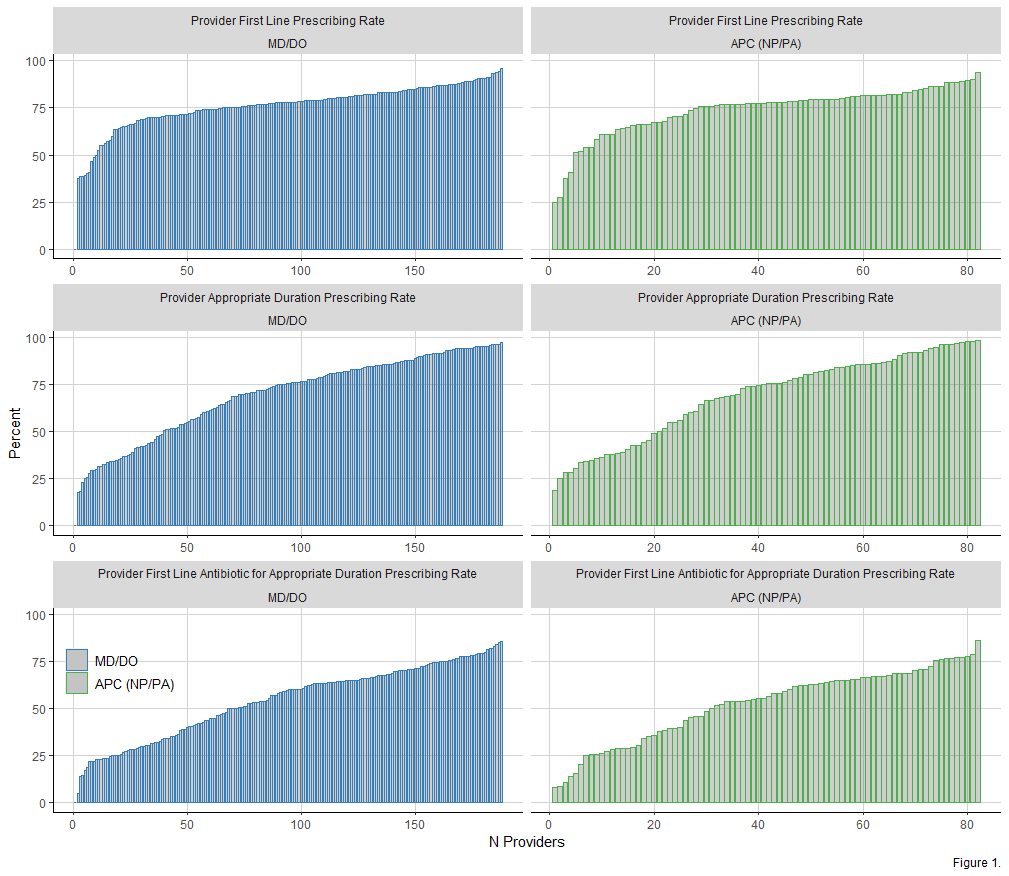


**Supplementary Figure 1.** Individual provider variability for MD/DO and APC (NP/PA) clinicians for first line antibiotic prescriptions, appropriate duration prescriptions, and first line antibiotic prescriptions for an appropriate duration. Both clinician types exhibited similar inter-provider variability for measured outcomes.
